# Performance analysis of Leica Biosystems p16 monoclonal antibody in oropharyngeal squamous cell carcinoma

**DOI:** 10.1186/s13000-025-01601-w

**Published:** 2025-01-24

**Authors:** Selvam Thavaraj, Max Robinson, Shubham Dayal, Claire Bowen

**Affiliations:** 1 Head and Neck Pathology, Centre for Clinical, Oral & Translational Science, Faculty of Dentistry, King’s College London Strand, Guy’s Campus, London, SE1 9RT UK; 2 Department of Oral & Maxillofacial Clinical Sciences, Faculty of Dentistry, University of Malaya, Kuala Lumpur, 50603 Malaysia; 3 Department of Cellular Pathology, Newcastle Upon Tyne Hospitals NHS Foundation Trust, Newcastle upon Tyne, UK; 4 Medical and Scientific Affairs, Leica Biosystems Richmond Inc. 5205 US, Highway 12, Richmond, IL 60071 US

**Keywords:** p16, Oropharyngeal carcinoma, LBS RTU p16, HPV

## Abstract

**Background:**

Head and neck squamous cell carcinoma (HNSCC) is the sixth leading cause of cancer death globally, with newly diagnosed oropharyngeal squamous cell carcinoma (OPSCC) cases rising to 54,000 in the US alone in the year 2022. Recently, human papilloma virus (HPV) infection was more prevalent in OPSCC patients than the traditionally known carcinogens such as tobacco or alcohol. HPV 16 is the most common causative HPV strain, which is found in 5–10% of HNSCC patients. HPV 16’s E6 and E7 oncoproteins bind and inactivate p53 and retinoblastoma (Rb) tumor-suppressing genes. This causes aberrant over-expression of the cell cycle inhibitor gene, p16, leading to tumorigenesis. Leica Biosystems (LBS) has developed a p16 antibody (6H12 clone) for qualitatively identifying the p16 protein in formalin-fixed paraffin-embedded (FFPE) tissue by immunohistochemical staining. This method comparison study tested the concordance rates between ready-to-use (RTU) LBS p16/LBS RTU p16 antibody and Roche Tissue Diagnostics (RTD) CINtec p16 Histology immunohistochemical (IHC) assays by measuring overall agreement (OA), average positive agreement (APA), and average negative agreement (ANA) rates in 170 OPSCC FFPE cases. Interobserver agreement of the 2 assays and LBS RTU p16 comparison with the standard HPV molecular assays (DNA ISH and PCR) were also assessed.

**Methods:**

One hundred and seventy (170) unique oropharyngeal cancer cases were stained for qualitative analysis by the LBS p16 antibody on BOND III. This assay was compared to Ventana’s RTD E6H4 (CINtec) clone on Benchmark XT. A stained core was considered p16 positive if the Histoscore (H score) was ≥ 140 and negative if H < 140.

**Results:**

Across the pathologists, the agreement rate between the 2 assays ranged from OA, 98.7 – 98.8%, ANA, 98.8 -98.9%, and APA, 98.6%. For LBS RTU p16, the interobserver agreement was OA, 98.7%, ANA, 98.8%, and APA, 98.6%; while for RTD CINtec p16 assay, the concordance was OA, 98.7%, ANA, 98.8% and APA, 98.6%. In comparison to the HPV molecular testing, DNA ISH, and PCR, across pathologists, LBS p16 clone (LBS RTU p16) showed a concordance rate of 85.8-86.9% and 87.6-88.8%, respectively.

**Conclusion:**

LBS p16 monoclonal antibody demonstrated high concordance with CINtec p16 IHC assay across all the endpoints, suggesting a potential use of LBS RTU p16 clone in detecting p16 protein in oropharyngeal cancer cases.

## Background

Head and neck cancer is the sixth most common cancer type, leading to around 900,000 new diagnoses and 450,000 (4.5%) of cancer-related annual deaths globally [1]. The rise in the global incidence of this disease is largely accounted for by the increase in tumors originating in the oropharynx. The marked increase in oropharyngeal squamous cell carcinomas (OPSCC) over the past two decades is driven by high-risk types of human papillomavirus (HPV) [1]. Against this epidemiological background, there is now robust evidence that patients with HPV-associated OPSCC have better survival outcomes compared to site- and stage-matched HPV-independent OPSCC [2]. The consistent difference in prognosis has led to HPV-associated OPSCC now being staged according to different clinicopathological criteria compared to HPV-independent oropharyngeal cancers [3–7]. For these reasons, several professional bodies, including the National Comprehensive Cancer Network, National Institute of Health and Care Excellence, College of American Pathologists (CAP), and Royal College of Pathologists, mandate HPV testing in all OPSCCs [8–13].

Under normal physiological conditions, the tumor suppressor proteins, p53, and retinoblastoma (pRB) are responsible for balancing cell cycle progression. The p16 (p16^INK4a^) protein keeps the pRB protein in its inactivated/hypophosphorylated state, resulting in control of the cell cycle’s growth phase. However, in the presence of active high-risk HPV infection, the HPV E7 and E6 oncoproteins binds to and degrades pRB and p53, respectively, leading to uncontrolled cell proliferation and oncogenesis [14, 15]. In HPV-positive tumors, where pRB is degraded by the E7 protein, p16 is freed as it cannot engage with pRB, leading to the overexpression of this protein in cancer cells. Therefore, p16 overexpression, as assessed by immunohistochemistry (IHC), is clinically utilized as a surrogate marker for transcriptionally active high-risk HPV in OPSCC [15, 16]. Since p16 overexpression may rarely arise by HPV-independent mechanisms resulting in occasional false positive tests, some authorities suggest that a p16 positive result should be followed by HPV-specific testing such as DNA or RNA in situ hybridization (ISH) or reverse transcriptase polymerase chain reaction (RT-PCR) [17–20]. Nevertheless, due to sufficient reliability, widespread availability, and low costs, p16 IHC is generally accepted as a stand-alone test to determine HPV status for prognostication and staging in OPSCC [6, 7, 21, 22].

The LBS RTU p16 is an in vitro diagnostic (IVD) device intended for the qualitative identification of human p16 protein in FFPE IHC-stained tissues. While the Roche Tissue Diagnostics (RTD) CINtec p16 IHC assay has been approved (FDA Class II IVD device) as a surrogate marker for HPV in FFPE cervical biopsy samples on the BenchMark staining platform [23], there is no approved p16 IHC assay to determine HPV status in OPSCC tumor samples to date. In this context, it should be noted that p16 staining patterns are clone-dependent [24, 25] and the clinical utility of any given clone should be evaluated against high-risk HPV specific tests. This study, hence, compares the analytical performance of Leica Biosystems (LBS) developed monoclonal p16 antibody (clone 6H12) with the CINtec p16 (clone E6H4) assay in reference to the other commonly used assays, namely DNA ISH and PCR in OPSCC [26] cancer samples.

## Methods

### Patient samples and demographics

This method comparison study enrolled 170 OPSCC FFPE samples. The study cases were collected from Newcastle Hospitals NHS Foundation Trust Royal Victoria Infirmary (NUTH RVI) hospital over 10 years (2002–2011). To avoid selection bias, the first 17 FFPE cases for each year enrolled with the hospital were included as study cases (17 cases/year x 10 years) totaling 170 unique study specimens. Of this cohort, 140 were male, and the remaining 30 samples were from female patients, with a mean age of 59.3 ± 10.8 years (average ± standard deviation). The tumor sites were “tonsil” for 64.7% (110/170), “soft palate” for 9.4% (16/170), and “oropharynx (not otherwise specified)” for the remaining 25.9% (44/170) of the samples. A case was added to the study cohort based on the predefined study inclusion/exclusion criteria. All the samples provided by LBS to the study sites were included in the study. The testing was repeated if: any of the procedural controls failed, faults unrelated to the assay, such as human error, power failure, tissue lifted from the slide so that the slide read/case interpretation was not possible, or there was a deviation in the staining protocol for the tested tissue. Additionally, a case or core was excluded if there was an insufficient tumor sample or uninterpretable staining due to improper tissue fixation/processing of the original clinical sample.

### Study design

**Fig. 1:**
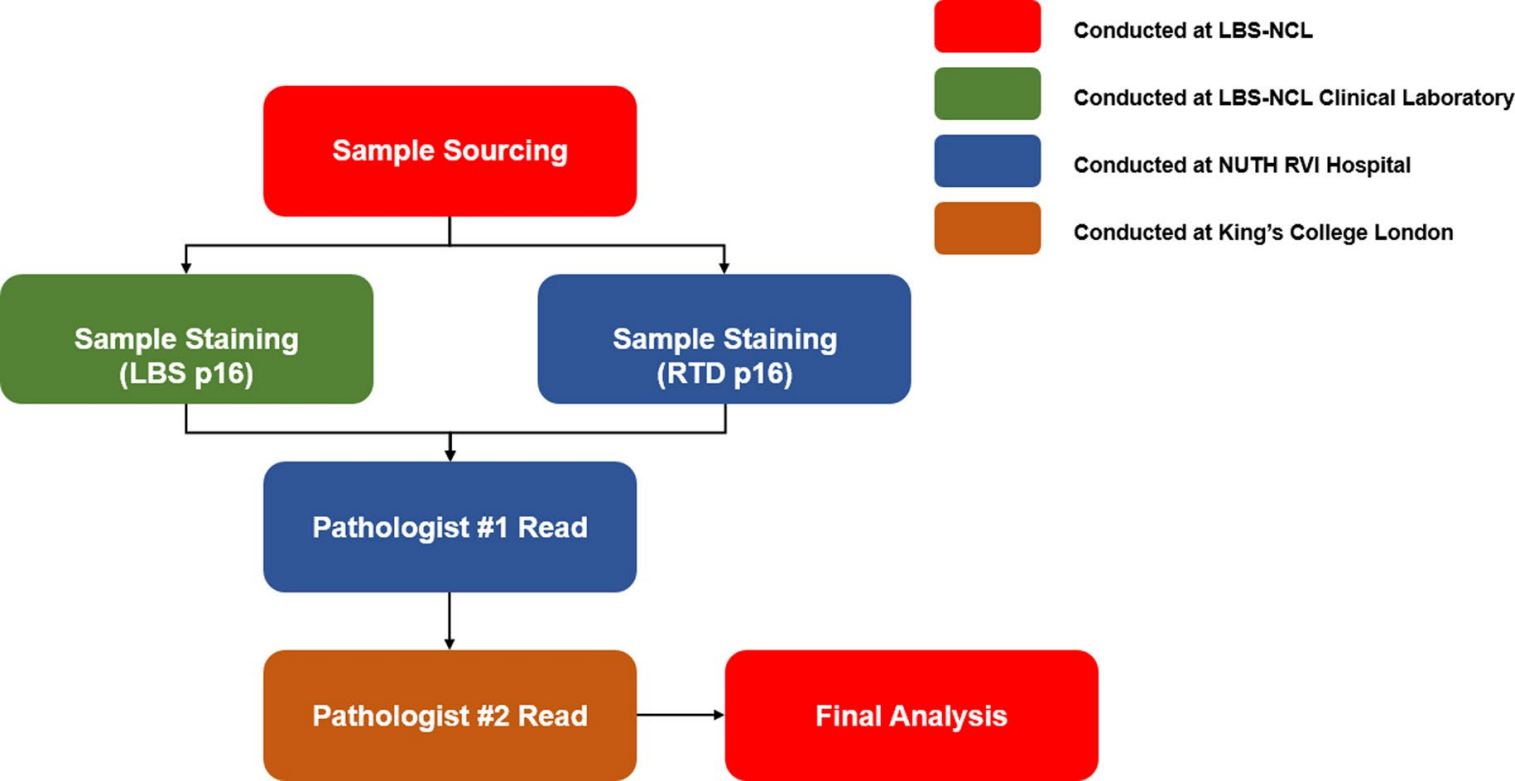
Study workflow describing the processing of the OPSCC cases and subsequent analysis of the investigational product (Leica Biosystems p16 antibody) and its comparator (Roche Tissue Diagnostics p16 antibody). LBS-NCL: Leica Biosystems-Newcastle, NUTH RVI: Newcastle Hospitals NHS Foundation Trust Royal Victoria Infirmary, OPSCC: Oropharyngeal squamous cell carcinoma

One hundred and seventy cases material was distributed in 10 separate tissue microarrays. Each tissue microarray contained three 1-mm-cores from each case FFPE material for a total of 51 embedded cores. Each tissue microarray was sectioned at 4 μm. Therefore, the study material consisted of 510 cores (51 cores/tissue microarray x 10 tissue microarray blocks). The 2 antibody assays were run on 2 separate investigational sites, with 1 site running the LBS p16 (LBS RTU p16) stained samples on LBS BOND-III while the other site ran RTD E6H4 (CINtec) stained samples on RTD Benchmark XT automated stainer (Fig. 1). Each site was pre-qualified before the study by LBS based on their experience running similar IHC assays and having appropriate resources and staff to conduct the study appropriately. Since the study only used deidentified FFPE tissue samples, this observational study was given an informed consent waiver by the Institutional Review Board (IRB) prior to study commencement.

Each of the 170 samples was stained by LBS and RTD antibodies on their respective automated stainer, BOND-III and Benchmark XT. Each TMA had its own normal tonsil tissue control. Additionally, each run was accompanied by positive and negative tissue control slides. Samples set stained with LBS RTU p16 and RTD CINtec p16 were read and scored by both pathologist 1 and pathologist 2 (Fig. 1).

Each unique case was given a Histoscore (H-score) based on the staining intensity of tumor cells. The H-score of a tumor sample was calculated by adding the product of tumor cell intensity (intensity graded as 0–3, where 0 through 3 grades are no staining, weak staining, median staining, and strong staining) and percent of tumor cells at that intensity grade. For example, an H-score of 250 was given to a tumor sample having 10% tumor cells with weak p16 staining (grade 1), 30% medium staining (grade 2), and the remaining 60% cells having strong staining (grade 3), and none expressing 0 grade or no staining (10% tumor cells x grade 1 + 30% tumor cells x grade 2 + 60% tumor cells x grade 3 + 0% tumor cells x grade 0 = H-score 250). An H-score of ≥ 140 was considered positive for p16, while an H-score < 140 was negative for p16 status.

All the cases were pre-characterized for HPV status by conducting DNA ISH and PCR on the study samples in a previous study conducted by Schache et al. [27] The pre-characterization was done by Qiagen Multiplex PCR Kit and DNA ISH by using RTD Inform HPV III Family 16 probe and Leica High-Risk HPV probe on their respective BenchMark and BOND-III automated staining instruments at pathology laboratories having Clinical Pathology Accreditation (CPA) certification. The sample pre-characterization using Leica DNA ISH assay was performed in the current study by one of the reading pathologists.

### Study objectives and endpoints

The primary objective of this non-interventional study was to compare (17 cases x 10 FFPE TMAs = 170 measurements) the analytical performance of the 2 assays (LBS RTU p16 and RTD CINtec p16). A secondary objective was to determine the interobserver agreement between 2 specialist head and neck pathologists (17 cases x 10 FFPE TMAs = 170 measurements/reader) for each assay, while an exploratory objective was to evaluate how each assay compares with HPV DNA ISH and PCR tests.

The endpoints for the primary objective were to estimate APA, ANA, and OA between the 2 assays. For the secondary objective, the endpoints were to estimate APA, ANA, and OA between the 2 reading pathologists for each assay. Lastly, for the exploratory objective of comparing both assays’ HPV DNA ISH and PCR test results, the endpoints, APA, ANA, and OA between the 2 assays were measured.

For the primary objective analyses, in addition to calculating the average H-Score, a majority call analysis and an individual core analysis were performed. For the majority call analysis, instead of taking the average of the triplicate H-scores, the majority H-score (most frequent positive or negative H-score in 3 cores of a sample) was captured (17 cases/TMA x 10 TMAs = 170 H-score measurements) to assign a specimen as either HPV negative (H-score < 140) or positive (H-score ≥ 140). For individual core analyses, an H-score of each core was estimated [17 cases/TMA x triplicates (3)/case x 10 TMAs = 510 measurements)] and compared for tested and the comparator assays. Similarly, inter-reader concordance cases (170 unique cases) and core-wise measurements (510 cores) were captured.

### Statistical analysis

For primary, secondary, and exploratory objectives, APA, ANA, and OA were estimated using the SAS software 9.4. Since neither of the assays (LBS RTU p16 or RTD CINtec p16) are considered standard reference IHC tests, we estimated the agreement rates by calculating the average positive (APA) and negative agreements (ANA).

To calculate these end-point values, a non-parametric bootstrap method of 5000 samples were used to calculate the 2-sided 95% CI between 2.5th -97.5th percentile sample distribution.

Samples excluded from the study were removed from the final data analysis as these met the predefined exclusion criteria as detailed above.

## Results

### LBS RTU p16 and RTD CINtec p16 had comparable immunostaining and H-score agreement rates

Out of 170 cases, 156 and 162 were deemed evaluable by Pathologist/Reader 1 and Pathologist/Reader 2, respectively. Fourteen specimens were excluded by Reader 1, while 8 specimens were excluded by Reader 2 as not containing tumor or histologically uninterpretable. The OA between the 2 assays, as estimated by Pathologist 1, was 98.7%, while Pathologist 2’s OA was 98.8%. A detailed analysis of each endpoint is provided in Table 1.

**Table 1:** Agreement rate measurement between LBS RTU p16 and RTD CINtec p16

Subgroup	*N*	APA		ANA		OA	
		Estimate	95% CI	Estimate	95% CI	Estimate	95% CI
**Reader 1**	156	98.6%	[96.3–100%]	98.8%	[96.9–100%]	98.7%	[96.8–100%]
**Reader 2**	162	98.6%	[96.4–100%]	98.9%	[97.1–100%]	98.8%	[96.9–100%]

a. Fourteen and 8 cases were excluded by Reader 1 and 2, respectively due to the uninterpretable staining of any of the triplicate cores because of insufficient tumor, insufficient tissue fixation or sample processing

b. Percent agreements and 95% CI was produced using percentile bootstrapping approach

Additionally, assay sensitivity was analyzed by measuring the majority H-score (positive or negative) amongst triplicate cores/case instead of the average H-score. For each case, a majority score was considered. Therefore, a total of 170 measurements (17 unique cases/TMA x 10 TMAs) were recorded. There were no changes in the Reader 1 analyses compared to the average H-scores for any of the assays, while for Reader 2 for 1 case, there was a different outcome for the RTD CINtec p16 assay (the reading for the majority analysis was negative instead of positive when estimated via average H-score) while no changes were reported between the majority and average H-score for the LBS RTU p16 assay.

The sensitivity was also analyzed by measuring the H-scores of each of 510 cores (17 unique cases/TMA x 3 cores/case x 10 TMAs). One hundred and twenty-eight and 123 cores were excluded by Reader 1 and Reader 2 due to uninterpretable staining, respectively. A pairwise H-score measurement analysis is given in Table 2.

**Table 2:** TMA core-wise sensitivity analysis between LBS RTU p16 and RTD CINtec p16

Subgroup	*N*	APA		ANA		OA	
		Estimate	95% CI	Estimate	95% CI	Estimate	95% CI
**Reader 1**	382	98.9%	[97.6–99.7%]	99.0%	[98.0–99.8%]	98.9%	[97.8–99.7]
**Reader 2**	387	97.8%	[96.0–99.2%]	98.1%	[96.6–99.3%]	97.9%	[96.4–99.2%]

a. Percent agreements and 95% CI was produced using percentile bootstrapping approach

Overall, the endpoint measurements across the standard average score method, case-wise majority score method, and core-wise agreement measurements were similar.

### LBS RTU p16 and RTD CINtec p16 demonstrated similar inter-reader agreements

**Fig. 2:**
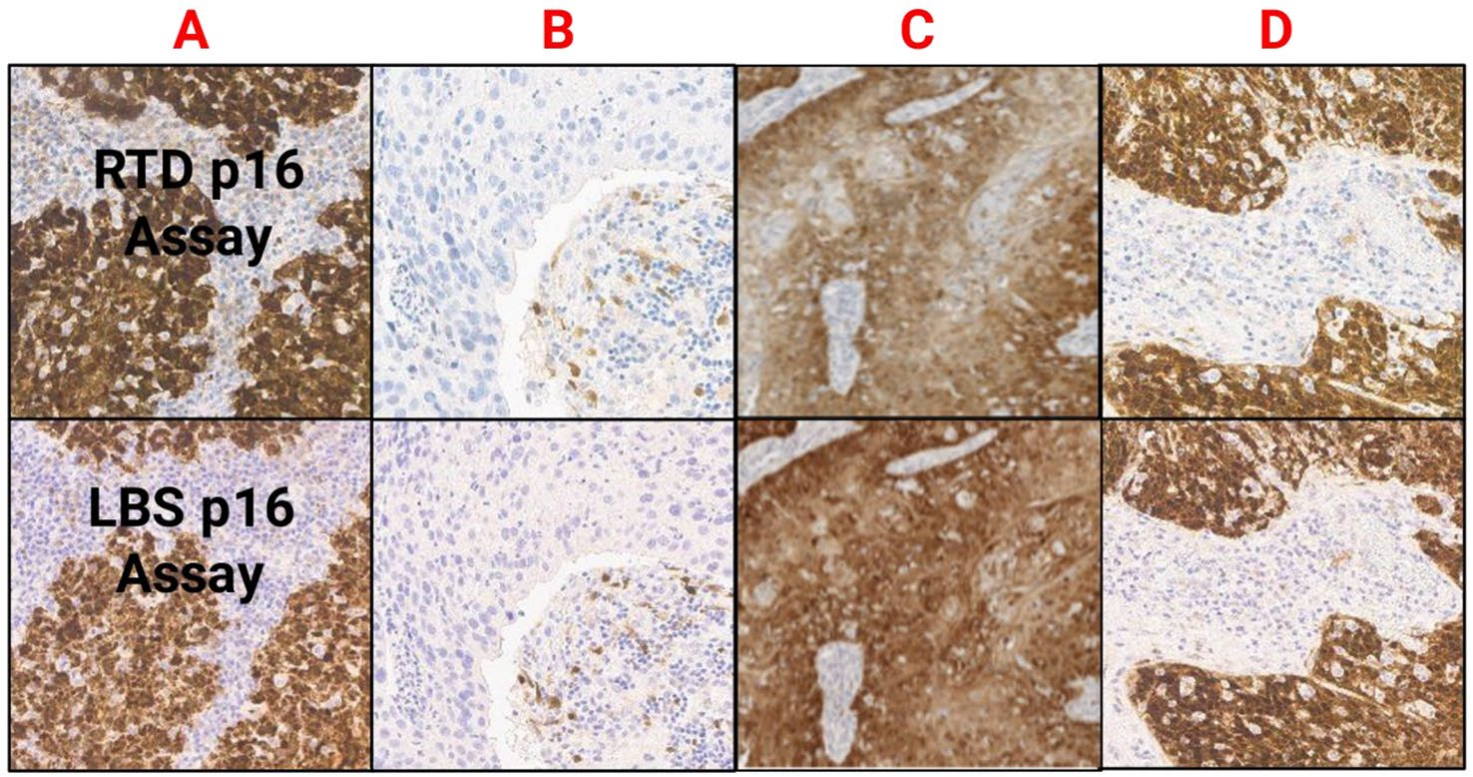
Comparative images of positive OPSCC cases (**A** , **D**) with strong, diffuse p16 staining in > 70% of tumor cells (H-Score ≥ 140). Image **B** shows a negative OPSCC case, with focal p16 staining in < 70% tumor cells (H Score < 140). Image **C** shows an HPV positive tumor (as confirmed by DNA ISH and PCR), but which was equivocal when assessed with RTD assay and positive with LBS assay

The inter-reader agreement rates by cases and by cores were also evaluated. The inter-pathologist/reader OA for the LBS p16 assay was 98.7% by each case and 98.9% by each core, while for the RTD p16 assay the OA by each case and core was 98.7% and 99.5%, respectively. A detailed analysis is mentioned in Table 3. Examples of positive concordant, negative concordant and discordant scores are illustrated in Fig. 2.

**Table 3:** Inter-reader agreement rate between LBS RTU p16 assay and RTD CINtec p16 assay

Inter-reader agreement rate for LBS RTU p16 Assay							
Subgroup	*N*	APA		ANA		OA	
		Estimate	95% CI	Estimate	95% CI	Estimate	95% CI
**Unique Cases**	157	98.6%	[96.3–100%]	98.8%	[97.0–100%]	98.7%	[96.8–100%]
**Individual Cores**	375	98.9%	[97.6–99.7%]	99.0%	[97.9–99.8%]	98.9%	[97.8–99.7%]
**Inter-reader agreement rate for RTD CINtec Assay**							
**Subgroup**	**N**	**APA**		**ANA**		**OA**	
		**Estimate**	**95% CI**	**Estimate**	**95% CI**	**Estimate**	**95% CI**
**Unique Cases**	160	98.6%	[96.4–100%]	98.8%	[97.0–100%]	98.7%	[96.8–100%]
**Individual Cores**	396	99.5%	[98.6–100%]	99.5%	[98.8–100%]	99.5%	[98.7–100%]

a. Percent agreements and 95% CI was produced using percentile bootstrapping approach

### LBS RTU p16 and RTD CINtec p16 showed similar performances when compared to HPV DNA ISH and PCR

Each reader’s analysis assay results were compared to the high-risk HPV DNA ISH and PCR pre-characterized HPV status. For each IHC assay, pairwise comparisons were made to the ISH and PCR tests (ISH or PCR’s HPV positive or negative status vs. IHC p16 positive status if H-score is ≥ 140 or negative if H-score is < 140).

For the LBS p16 assay, the OA for Reader 1 LBS HPV DNA ISH, RTD HPV DNA ISH, and PCR ranged between 85.8 − 93.0%, while for Reader 2, the OA for the same analysis ranged between 86.9 − 93.2%. For the RTD p16 assay, the OA for Reader 1 LBS HPV DNA ISH, RTD HPV DNA ISH, and PCR ranged between 86.0 − 93.2%, while for Reader 2, the OA for the same analyses ranged between 87.0 − 94.5%. A detailed analyses is described in Table 4.

**Table 4:** Comparative analysis of LBS RTU p16 and RTD CINtec p16 assays with HPV DNA ISH and PCR

LBS p16 IHC assay comparison with high-risk HPV DNA ISH and PCR							
Subgroup	*N*	APA		ANA		OA	
		Estimate	95% CI	Estimate	95% CI	Estimate	95% CI
**Reader 1 with LBS HPV DNA ISH**	156	82.6%	[75.2–89.2%]	87.9%	[82.6–92.5%]	85.8%	[80.3–91.1%]
**Reader 1 with RTD HPV DNA ISH**	157	91.5%	[86.0–96.2%]	93.9%	[90.1–97.3%]	93.0%	[88.7–96.8%]
**Reader 1 with PCR**	155	87.6%	[81.6–92.7%]	87.6%	[81.7–92.7%]	87.6%	[82.4–92.6%]
**Reader 2 with LBS HPV DNA ISH**	160	84.1%	[76.9–90.5%]	88.7%	[83.4–93.3%]	86.9%	[81.4–92.0%]
**Reader 2 with RTD HPV DNA ISH**	162	91.9%	[86.8–96.3%]	94.1%	[90.2–97.3%]	93.2%	[89.0–96.9%]
**Reader 2 with PCR**	160	88.9%	[83.4–93.6%]	88.6%	[82.9–93.4%]	88.8%	[83.8–93.4%]
**RTD p16 IHC assay comparison with high-risk HPV DNA ISH and PCR**							
**Subgroup**	**N**	**APA**		**ANA**		**OA**	
		**Estimate**	**95% CI**	**Estimate**	**95% CI**	**Estimate**	**95% CI**
**Reader 1 with LBS HPV DNA ISH**	157	83.0%	[75.7 -89.6%]	87.9%	[82.8 -92.7%]	86.0%	[80.4 -91.2%]
**Reader 1 with RTD HPV DNA ISH**	160	92.0%	[86.6 -96.3%]	94.0%	[90.2 -97.3%]	93.2%	[89.0 -96.9%]
**Reader 1 with PCR**	158	88.2%	[82.6 -93.0%]	87.7%	[82.0 -92.6%]	88.0%	[82.8 -92.6%]
**Reader 2 with LBS HPV DNA ISH**	161	83.9%	[76.5 -90.2%]	89.0%	[83.9 -93.4%]	87.0%	[81.5 -91.9%]
**Reader 2 with RTD HPV DNA ISH**	164	93.4%	[88.5 -97.4%]	95.2%	[91.9 -98.1%]	94.5%	[90.7 -97.6%]
**Reader 2 with PCR**	162	88.8%	[83.3 -93.6%]	88.8%	[83.5 -93.6%]	88.9%	[84.0 -93.5%]

a. Percent agreements and 95% CI was produced using percentile bootstrapping approach

## Discussion

p16 is now an accepted surrogate marker for HR-HPV detection in OPSCC [11]. With the improved prognosis of HPV-positive cases and recommendations by guidelines to test p16 status via IHC in these tumours, it has become imperative to have a reliable method to test the p16 protein status in these samples. This method comparison study demonstrated that LBS RTU p16 monoclonal antibody has comparable analytical equivalence to RTD p16 CINtec antibody for detecting p16 protein in OPSCC samples. HPV-related OPSCC has a better prognosis; however, currently, there is no single approved test/assay that could precisely detect OPSCC status in an individual. As mentioned before, p16 IHC analysis is recommended by several accredited societies to estimate the high-risk HPV status. However, there is currently no FDA-approved p16 IHC assay/IVD device to determine HPV status in OPSCC cases. The only approved p16 IHC test is RTD CINtec which only applies to cervical carcinoma tumor types. Since CINtec is the only approved p16 IHC assay, we, therefore, compared the LBS RTU p16 and RTD CINtec p16 performance in OPSCC FFPE samples. Likewise, since there is no approved IHC test for the tested indication, agreement rates were determined by estimating the concordance of the 2 assays as read by the 2 pathologists.

Previous studies have used cross-staining systems, i.e., using a specific clone on a different staining platform and vice versa [24, 28]. In contrast to these studies, the current non-interventional study used manufacturer-recommended IHC staining for both the assays, including the use of the same manufacturer’s antibody and staining platforms, thereby minimizing different staining system-related variability. Few studies have directly compared p16 antibodies’ performance in OPSCC. Shelton et al. compared E6H4 (CINtec), JC8 (Santa Cruz Biotech), and G175-405 p16 (BD Biosciences) IHC clone clinical performance in OPSCC TMAs; out of these clones CINtec was the best-performing antibody [24]. In the current study, LBS RTU p16 and RTD CINtec p16 produced comparable staining and inter-observer variability, indicating LBS RTU p16’s optimal and reproducible epitope labelling capabilities.

Although p16 can be used in isolation, some authorities recommend a second-tier HPV-specific test in p16 positive cases [10–13]. Therefore, we also compared the LBS RTU p16 protein status with HR-HPV DNA ISH and PCR of the same sample. The OA rate, as analyzed by both the study pathologists, was around 90.0% and 88.0% for DNA ISH and PCR, respectively, which was comparable to the performance of RTD’s CINtec assay (Table 4). Due to variation in the sensitivity and specificity of DNA ISH and PCR, there are no predefined acceptance criteria percentage to compare IHC assays. Therefore, the qualitative performance of the LBS and RTD assays could not be determined. Since neither of the assays is considered a ‘reference test’, including the DNA ISH and PCR; [26, 29] we, therefore, calculated agreement rates (ANA and APA) between assays, which can give an idea of the assay’s performance relative to the other tested assays.

As described in Table 4, the relative specificity (ANA) for both the assays with respect to the ISH and PCR measurement ranged from 87.7 to 94.1% (LBS p16 IHC assay 87.9-94.1% and RTD p16 assay 87.7-94.0%) and the relative sensitivity (APA) was between 82.6 − 93.4% (LBS p16 IHC assay 87.9-94.1% and RTD p16 assay 87.7-94.0%) which is in accordance to the current literature reported read-outs [16, 20, 30–32].

In the current non-interventional study H-score- although a newer scoring method, has been validated and used by other studies [33, 34] provided a good estimation of each sample’s p16 status, although it would have been interesting to observe how these H-scores correlate with the standard 70% p16 positivity cut-off [21]. Inter-reader variability is a common concern for any histologic assay [35, 36]. The inter-reader reproducibility between the assays for both the pathologist in the current study was close to 99.0% (OA for the first pathologist was 98.7% while for the second pathologist was 98.8%), indicating low variability and high reproducibility thereby suggesting that the LBS RTU p16 assay has a potential to be a reliable prognosticator for OPSCC by determining the p16 status. Additionally, the prevalence rate of HPV infection for these samples, as estimated by Schache et al. [27] was 44.1%, which is similar to the global rate of 44.8% [37], indicating the potential clinical utility of the LBS RTU p16 as its relative sensitivities and specificities were similar to all the tested assays.

The study had some limitations. The clinical outcome of the tested clone was not analyzed by performing a well-designed clinical trial that may have included increased sample size leading to more case reads and study pathologist generating more comparative reads. Endpoints such as overall survival rate determination or patient stratification via p16 IHC determination would be a true evaluation of the clinical utility of the assay. Additionally, the analytical performance (sensitivity and specificity) of LBS RTU p16 was not tested against statistically driven acceptance criteria. Also, the current study did not analyze how the LBS assay compared to RTD assay for borderline/difficult cases where the average H score is around 140, having a low HPV burden.

While the results of the current study are encouraging, it is important to note that these results are preliminary, and this study does not claim that the LBS RTU p16 assay can be used as a laboratory-developed or diagnostic test for HPV infection status in OPSCC cases. Such claims would require review and approval by an accredited agency, following which analyzing its clinical utility in a well-designed clinical trial could lead to the clone’s regulatory approval.

Detection methods such as estimating circulating HPV-DNA are also being tested, but the technique still requires further investigation [38] and is also challenging to execute compared to the p16 IHC detection method. Additionally, novel algorithms are being developed that would aid pathologists in accurately detecting p16 in OPSCC by identifying other morphological features, such as nuclear density and size [22]. However, these artificial intelligence (AI)-based developments are yet to be tested in clinical settings. Future studies may utilize the testing of the LBS RTU p16 clone and an AI-based model in a clinical outcome-based trial to improve quantitative analysis of the p16 protein. The test clone, in the future, can also combine these novel algorithms to refine the p16 status, thereby improving patient care and post-operative quality of life.

Based on the current observational study this LBS clone can potentially fill a gap in early and accurate detection of HPV in OPSCC. Although the study results look promising for the identification of disease etiology, further investigations, particularly the assay’s potential of not to over diagnose a test cohort remains to be seen. Therefore, a clinical study with sufficient statistical rigor is warranted to confirm the results from the current study and to explore the LBS RTU p16 clone’s clinical utility in providing timely and appropriate treatment to OPSCC patients.

## Conclusion

The LBS RTU p16 clone and RTD CINtec p16 produced comparable results for the detection of p16 status in OPSCC samples, demonstrating LBS p16 clone’s (LBS RTU p16) potential to be used as a surrogate marker for high-risk HPV in clinical medicine.

## Data Availability

No datasets were generated or analysed during the current study.
